# Outdoor thermal comfort in courtyard-shaped housing projects: a simulation study for a tropical region

**DOI:** 10.1007/s00484-026-03208-2

**Published:** 2026-04-28

**Authors:** B. S. Apolinário, E. Masiero, E. Krüger

**Affiliations:** 1 Programa de Pós-Graduação em Engenharia Urbana, Universidade Federal de São Carlos (UFSCar), São Carlos, Brazil; 2 Programa de Pós-Graduação em Engenharia Civil, Universidade Tecnológica Federal do Paraná (UTFPR), Curitiba, Brazil

**Keywords:** Microclimate, Courtyard Buildings, Thermal Stress, Social Housing

## Abstract

Inadequate thermal conditions in social housing projects (SHP), combined with global warming, increased frequency of heatwaves, and energy poverty, intensify health problems related to thermal stress. Addressing this issue involves exploring microclimate-improving solutions, such as courtyard buildings. Current research mainly focuses on fully enclosed courtyards, with limited analysis of their use in SHP. This study aims to assess the thermal benefits of courtyard building types on the microclimate and evaluate their suitability for social housing. Microclimatic simulations using ENVI-met analyzed courtyard buildings with heights of 14 and 29 m and ground-floor openings. The results show that during heatwaves, periods of extreme thermal stress were reduced, with PET decreases reaching up to − 11.7 K, while nighttime increases remained minor. On non-heatwave days, courtyard buildings lowered daytime heat stress and decreased nighttime cold stress. Vertical temperature profile analysis revealed that ground-floor openings improved ventilation and decreased heat buildup at that level, while mid-level floors experienced higher heat concentration due to limited sky exposure and reduced wind speed. These findings support the use of courtyard buildings in SHP and highlight the thermal benefits of ground-floor openings, including improved ventilation and reduced health-related hazards for occupants.

**Supplementary Information:**

The online version contains supplementary material available at 10.1007/s00484-026-03208-2.

## Introduction

In several countries, particularly in the Global South, social housing often lacks adequate thermal conditions, which contribute to the occurrence and exacerbation of heat-stress-related health problems (Porras-Salazar et al. 2020; Kurmanbekova et al. 2024). This situation is further intensified by global warming and the increasing frequency of heatwaves. Additionally, energy vulnerability arises when economic constraints limit access to air conditioning or electricity (Desvallées 2022; Simões and Leder 2022).

In response to these challenges, numerous studies have examined thermal improvement strategies for building envelopes (Gabriel et al. 2024a, b; Oliveira and Alves 2021). However, the urban microclimate directly influences internal thermal conditions through interactions between external and internal environments (Givoni 1998). The influence of microclimate within social housing complexes (so-called housing projects), however, remains insufficiently explored.

Building geometry is a key factor influencing the microclimate, as it affects exposure to solar radiation, ventilation, and heat exchange (Zamani et al. 2018). Among various typologies, courtyard buildings are notable for their capacity to modulate solar radiation gain, ventilation, evaporative cooling, and thermal damping through their geometric configuration (Zhou et al. 2025). High occupancy coefficients and compact geometries can decrease external thermal comfort during cold periods and enhance it during hot periods by increasing shading (Martinelli and Matzarakis 2017; Natanian et al. 2019). Additionally, the energy performance of courtyard buildings depends on their proportions: square courtyards may be more efficient in certain climates, while deep, narrow courtyards or those with a low length-to-depth ratio offer distinct advantages based on local thermal requirements (M’Saouri El Bat et al. 2023; Tabadkani et al. 2022).

In Brazil, while studies on residential courtyard buildings are lacking, research on public courtyards in semi-humid tropical climates demonstrates a reduction in external thermal discomfort for much of the year, with outcomes varying according to solar radiation patterns and courtyard morphology (Callejas et al. 2020; Callejas and Krüger 2022).

The literature typically examines courtyard buildings as structures that enclose the courtyard at ground level, creating fully enclosed spaces (Diz-Mellado et al. 2023; Han et al. 2023; Hao et al. 2019; Zhang et al. 2024). While this configuration offers sun protection and a sense of shelter, it may restrict ventilation and reduce both physical and visual connectivity between the courtyard and the surrounding urban environment. These limitations can negatively affect environmental comfort and the social use of the courtyard, particularly in climates where air circulation is essential.

Alternative courtyard geometries, such as L- or U-shaped floor plans, can reduce adverse effects by providing lower shading levels and lower building density. A potentially effective alternative is to adopt courtyard buildings with two open sides, located only on the ground floor, to create ventilation corridors and expand spatial connectivity, thereby strengthening the courtyard’s collective use and social function. This issue was analyzed by Sözen and Oral (2019), who reported a reduction of up to 8.6 °C in the PET index compared to courtyards without openings, highlighting the potential of this strategy. However, this study was limited to the hot summer Mediterranean climate (Csa).

Existing studies on the impact of courtyard buildings on the microclimate show potential benefits but do not clarify how geometric parameters influence microclimate and thermal performance during summer, as noted by Zhou et al. (2025). More research is needed on courtyards with ground-floor openings, especially in mostly warm climates. These gaps are particularly important, as official social housing programs in Brazil currently use only single-story, linear, or H-shaped designs (Ministry of Regional Development, 2020 apud Bavaresco et al. 2021), with no consideration of courtyard building designs.

The Brazilian housing deficit is estimated at approximately 6.2 million units (Fundação João Pinheiro 2022). The majority of this deficit (over 80%) is concentrated in the north, northeast, southeast, and mid-west regions, which are predominantly characterized by tropical climates (Koeppen’s Aw, Af, Aw), with a significant semi-arid area in the northeast (BSh). In these regions, mitigating heat gains in low-income housing remains a critical challenge.

Brazilian researchers have proposed various methods to improve the thermal performance of social housing buildings. Main strategies include enhancing the building envelope with better thermal insulation, advanced windows and glazing, controlled infiltration rates, and exploring alternative construction systems such as green walls and roofs (Dalbem et al. 2019; Gabriel et al. 2024a, b; Tubelo et al. 2018). Benincá et al. (2023) studied how building orientation and shape affect performance in Passo Fundo, Rio Grande do Sul, a region with a cold climate (Cfa), comparing H-shaped buildings to linear layouts. Their results show that a more compact design and less solar exposure in H-shaped buildings can reduce cooling needs. Gonçalves et al. (2024) investigated how building thermal performance interacts with microclimatic changes caused by vegetation and terrain permeability. They used an EnergyPlus model with a modified climate file that includes microclimatic data from ENVI-met simulations of different climate mitigation strategies. Cunha et al. (2025) evaluated how urbanization levels, as defined by the Local Climate Zone (LCZ) scheme (Stewart and Oke 2012), influence the thermal performance of social housing units. Together, these studies highlight the importance of managing building density to improve the thermal and energy efficiency of social housing.

Although this research area is significant, the influence of urban morphology indices on thermal comfort and energy demand in social housing units remains insufficiently studied. Variables such as street orientation, occupancy rate, proportion of permeable areas, building heights, sky view factors, spacing between buildings, and utilization coefficients require further investigation, particularly within the context of Brazilian climates.

Therefore, this study is justified to enhance understanding of the thermal, ventilation, and comfort aspects of courtyard building designs, with the aim of future implementation of such layouts in Brazilian social housing. The focus is on courtyard design, but the simulation model is limited to a simple courtyard shape that could later be improved with advanced parameterization, such as using cloud computing. A heatwave event during field monitoring provided an opportunity to evaluate the advantages and limitations of the courtyard shape under more extreme conditions.

## Method

This study employs experimental procedures that include computer simulations and the collection of microclimatic data in an urban social housing context. The methodology comprises six stages: (i) defining the study area; (ii) collecting microclimatic data; (iii) characterizing the days selected for simulation; (iv) calibrating the ENVI-met model; (v) modeling and simulating urban scenarios featuring courtyard buildings; and (vi) analyzing the results.

### Study site

The study was conducted in a recently developed single-family social housing complex in São Carlos, São Paulo State. São Carlos is situated at an altitude of 856 m a.m.s.l., between 47°30’ and 48°30’ west longitude and 21°30’ and 22°30’ south latitude. The housing complex covers approximately 508,047 m² and comprises 954 plots in the city’s peripheral area.

**Fig. 1:**
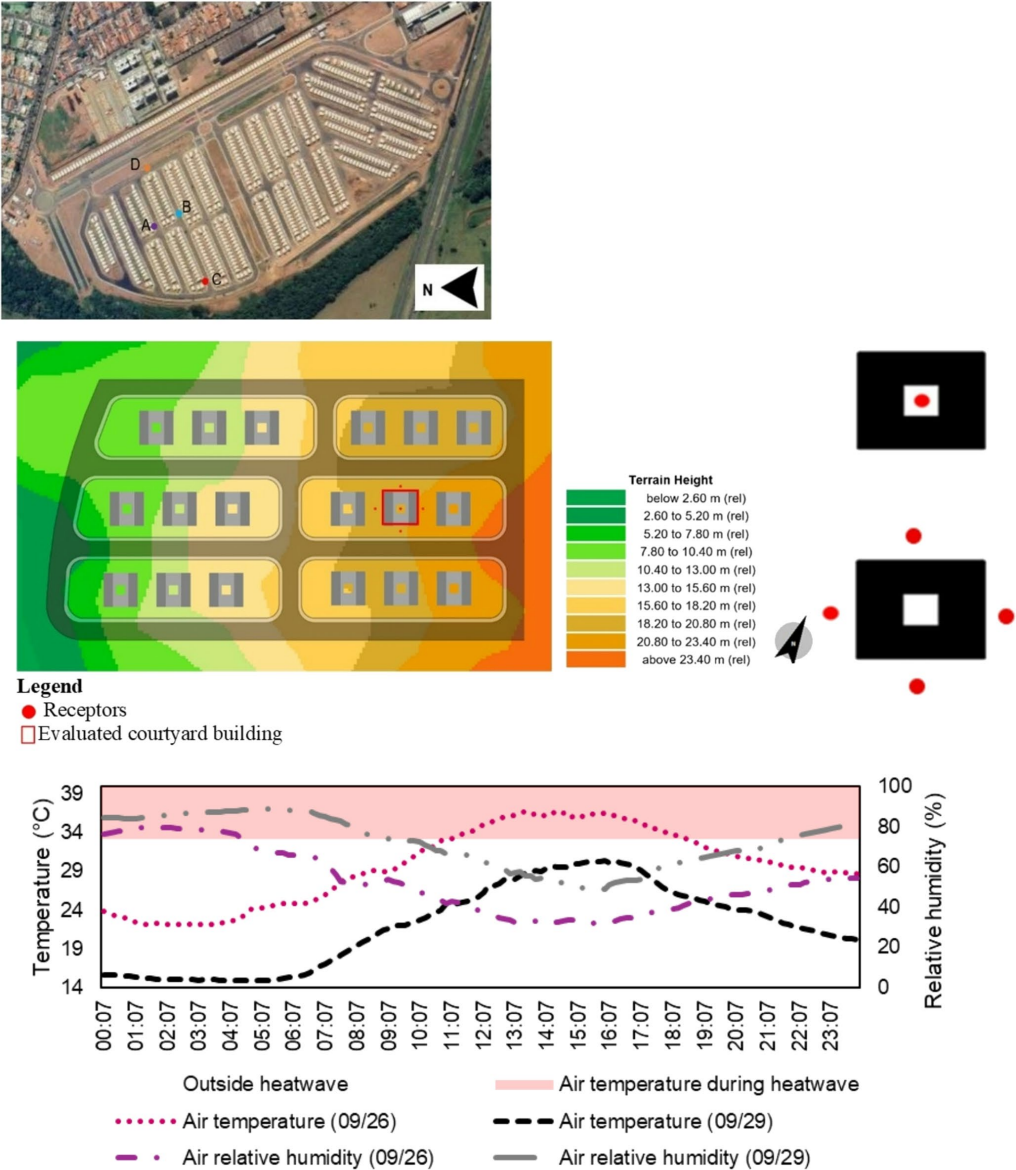
Study area, microclimatic data collection points, and temperature and air humidity on September 26 and 29 at the study site

According to the Köppen-Geiger classification, São Carlos has a subtropical climate with dry winters and hot summers (Cwa). Temperature data indicate that the highest temperatures occur between September and October, and the lowest between May and August. Figure 1 **(Supplementary materials)** shows variations in dry-bulb temperature and the classification of thermal stress based on the calculated Universal Thermal Climate Index (UTCI; https://www.utci.org/cost.html), considering scenarios with and without solar radiation and with wind, from the climate file BRA_SP_Sao.Carlos.868450_TMYx.2009–2023, for latitude 21° 58.80’ S, longitude 47° 53.02’ W, at 865 m a.m.s.l. (Lawrie and Crawley 2022). To that end, we used the CBE Clima Tool, version 0.9.0 (Betti et al. 2023), to illustrate mitigation scenarios for urban thermal conditions driven by building morphological aspects. Developed at the Center for the Built Environment, UCLA, Berkeley (https://clima.cbe.berkeley.edu/), the tool enables analysis and visualization of climate data, specifically EPW files from approximately 30,000 locations worldwide, sourced from two online data repositories: Energy Plus and Climate.One.Building.org. In one of Clima Tool’s data output modules, called “Data Explorer”, it is possible to run basic outdoor comfort scenarios, quantified as the UTCI thermal stress data, considering the original climate data modified for exposure options such as “no Wind”, “no Sun”, and “no Wind and no Sun”.

When inserting the climate file “BRA_SP_Sao.Carlos.868450_TMYx.2009–2023”, referring to São Carlos, created from data from the meteorological station whose international code (WMO) is 868,450, the four situations mentioned were considered. Figure 1 **(Supplementary materials)** presents the thermal stress categories for a base case without alterations to the surrounding morphology (reference) and for a shaded outdoor condition with wind, as in a patio configuration with large openings. Output data suggest that shading strategies have the potential to significantly reduce heat-related discomfort, eliminating almost all hours of extreme, very strong, and strong thermal stress throughout the year. With this in mind, the study examines the implementation of buildings with internal courtyards as an architectural strategy for heat mitigation in social housing in São Carlos and other cities with comparable climates.

### Microclimate data collection

To ensure realistic representation, the ENVI-met model was validated against field microclimate data from September 19 to October 4, 2023, around the vernal equinox. Temperature and relative humidity in the urban area were measured using four HOBO Pro V2 U23-001 sensors, which recorded data at predefined collection points at 10-minute intervals, as illustrated in Fig. 1.

The World Meteorological Organization (WMO) defines a heatwave as a period of at least five consecutive days with daily maximum temperatures exceeding the monthly climatological normal by at least 5 °C (INMET 2023). According to this criterion, São Carlos experienced a heatwave from September 19 to 27, 2023. The highest daily maximum temperature occurred on September 25, reaching 37.6 °C, which is 9.4 °C above the monthly climatological normal of 28.2 °C, based on the 1991–2020 climatological normal from the INMET 83,726 meteorological station at 21°58’S, 47°52’W, 860 m a.m.s.l. (INMET 2025). For the ENVI-met simulations, September 26 and 29 were selected to represent a typical heatwave day and a day outside the event, respectively. These dates correspond to the hottest and coolest days of the dry season. Temperature and relative humidity data recorded at point B are also shown in Fig. 1. On September 26, temperatures ranged from 22.1 °C to 36.9 °C, and relative humidity ranged from 32% to 80%. On September 29, temperatures ranged from 15.6 °C to 30.2 °C, and moisture ranged from 49% to 84%.

### Validation of ENVI-met

To validate the ENVI-Met model and verify its ability to represent the actual microclimate, an initial simulation was performed using the site’s existing urban morphology, characterized by single-family, single-story houses. The input data were entered into full forcing in the simulations. They included wind speed and direction (from the nearby weather station A711), air temperature and humidity (collected on site), and high, medium, and low cloud cover (from the INMET weather station 83726).

The validation was conducted by comparing the values ​​measured in the field with the simulated values ​​of air temperature and relative humidity, using the coefficient of determination (R²), the mean absolute error (MAE), the mean absolute percentage error (MAPE), and Willmott’s index of agreement (d) as relevant metrics.

**Fig. 2:**
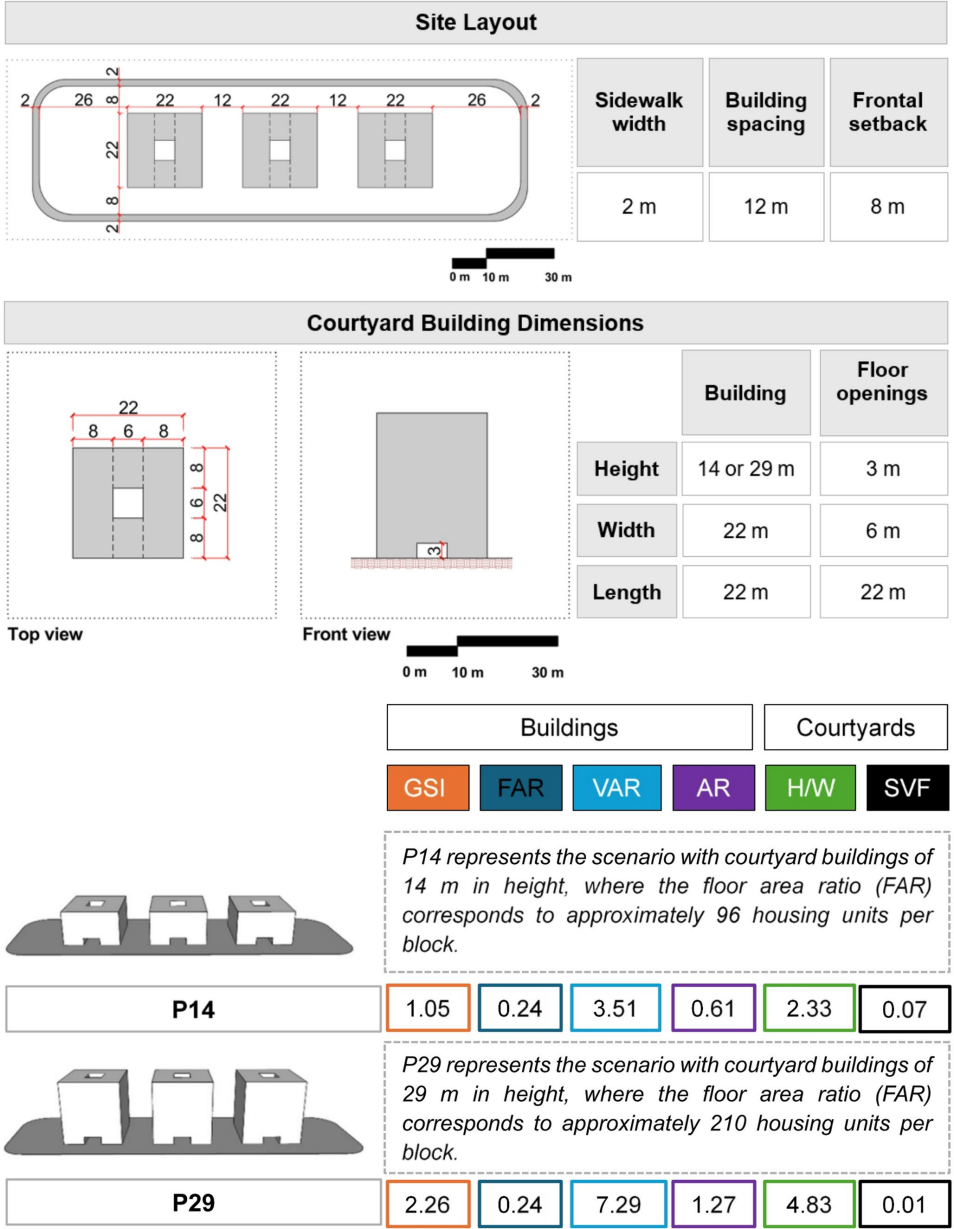
Dimensions and configuration of the courtyard building typologies, Simulation scenarios, and morphological indices

The results are presented in Fig. 2 **(Supplementary materials)**. For air temperature, R² = 0.989 and d = 0.995 were obtained, indicating high model reliability. The MAE (0.49) and MAPE (1.69%) values ​​reinforce this consistency. The same procedure was applied to air humidity, resulting in R² = 0.9879, d = 0.994, MAE = 1.52, and MAPE = 2.77%, values ​​that also confirm the adequate representation of the observed conditions. Thus, the simulation validation can be considered successful.

After validating the ENVI-met model, the housing typology was modified to courtyard buildings to assess their impact on the microclimate. The same full forcing configuration file used during validation was applied to all subsequent simulations, ensuring identical atmospheric boundary conditions across scenarios.

### Modeling and simulation of urban scenarios with courtyard buildings

The urban microclimate model was run using ENVI-met 5.6.1 (student version). This tool, based on computational fluid dynamics (CFD) and thermodynamic principles, simulates interactions among buildings, surfaces, soils, vegetation, and the atmosphere (Bruse and Fleer 1998). The student version provides full access to the software’s functionality, including the application of full forcing to meteorological boundary conditions and the generation of various output data for microclimate analysis, human thermal comfort, and pollutant dispersion. In our study, the main drawback of the ENVI-met student version is its limited parallel computing, which results in lower simulation speed.

In the full forcing configuration file, cloud cover values were used instead of direct radiation data. These cloud cover data reflect the conditions observed on the simulated days and were obtained from the meteorological records of the INMET station 83,726. In this setup, ENVI-met internally calculates solar radiation based on the cloud cover parameterization.

To assess the impact of courtyard building housing typology on the microclimate, urban scenarios with these buildings were simulated for a heatwave day and a day after this event, considering two building heights, 14 and 29 m, corresponding to four- and nine-story buildings, respectively. The dimensions and configurations of these buildings are presented in Fig. 2, alongside the urban morphology indices used for these scenarios. To identify the scenarios, acronyms were defined: P denotes the housing typology, H indicates “Heatwave”, and N represents “Non-heatwave”, i.e. a day outside the heatwave. To that end, a series of morphological indices was employed, as shown in Fig. 2.

Key morphological indices include the Gross Space Index (GSI), Floor Area Ratio (FAR), Volume-to-Area Ratio (VAR), Aspect Ratio (AR), Height-to-Width Ratio (H/W), and Sky View Factor (SVF). These morphological indices capture various aspects of urban density and spatial configuration. The Gross Space Index (GSI) measures building compactness as the ratio of built area to plot area. The Floor Area Ratio (FAR) quantifies land-use intensity by dividing gross floor area by plot area. The Volume-to-Area Ratio (VAR) assesses volumetric density as the ratio of building volume to plot area. The Façade-to-Lot Ratio (FLR) indicates vertical density by measuring the extent of vertical surfaces relative to the plot. The Aspect Ratio (AR) reflects a building’s compactness and is calculated as the building height divided by its length. The Height-to-Width Ratio (H/W) characterizes urban canyon geometry as the ratio of building height to street width. The Sky View Factor (SVF) represents the proportion of visible sky from a specific point.

Within the ENVI-met settings, the dimensions of streets, sidewalks, and buildings were adjusted to the nearest multiples of the grid values defined in the simulation. Table 1 presents these grid values, along with additional parameters and input data utilized in the simulations. The simulations employed a grid resolution of 2 × 3 m. This resolution was chosen to balance computational efficiency with sufficient spatial detail for accurately representing urban morphology and microclimatic processes relevant to the study. Employing a finer resolution, such as 1 × 1 m, would have significantly increased computational requirements and simulation time without yielding substantial improvements in the depiction of urban features pertinent to the research objectives. No further simplifications were applied to the model configuration beyond the selected grid resolution. All simulations used the identical boundary atmospheric conditions configuration file established during the initial model validation in Sect. 2.3. This approach ensured consistency across scenarios and enabled attribution of result differences solely to variations in urban morphology.

**Table 1:** ENVI-met simulation parameters

Parameter	Input data
*Simulation start (day before)* heatwave non-heatwave	25 Sept 2023 28 Sept 2023
*Day of analysis* heatwave non-heatwave	26 Sept 2023 29 Sept 2023
Total simulation time (h)	48
Meteorological background conditions	*Full forcing*
*Boundary conditions* Cloudiness Air velocity Wind direction Temperature and moisture	Weather station 83,726 Weather station A711 Weather station A711 Onsite measurements
Horizontal grids (m)	2
Vertical grids (m)	3
Height defined for telecospic factor (m)	60
Height of the domain (X-grids; Y-grids; Z-grids)	192; 119; 28
Size of the domain in m (X; Y; Z)	384; 238; 112.95
Albedo – building vertical surfaces	0.45
Albedo – building roof surfaces	0.30
Albedo – pavement (asphalt)	0.06
Albedo – sidewalks (concrete)	0.30
Albedo – bare soil	0.24

In addition to materials and surface characteristics, topographical data were obtained from the SRTM GL3 Arc_Sec database (NASA 2024), as provided by ENVI-met Monde. Upon completion of all configurations and modeling, the simulation model encompassed an area of 384 m by 238 m (Fig. 1).

To analyze the microclimatic data generated by the simulation, receptors were placed as shown in Fig. 1. Four receptors were placed around the building in accessible areas, such as sidewalks. The average of these four receptors was used to evaluate the thermal conditions outside the patio in four different solar orientations. Meanwhile, a fifth receptor was located at the center of the building’s courtyard to measure the microclimatic conditions within that space.

### Thermal comfort analysis

Thermal comfort was assessed using the PET index, which is calculated in ENVI-met 5.6.1 via the BIO-met module. The PET index (“Physiological Equivalent Temperature”, Höppe 1993) is grounded in the thermal balance of the human body. It quantifies the effects of the thermal environment on humans in terms of thermal stress or comfort, using the Munich Energy Balance Model for Individuals (MEMI, Höppe 1984). This model accounts for the body’s energy balance, including heat exchanges with the immediate environment, internal metabolic heat, and sensible heat produced by work (Höppe 1999). PET values are expressed in °C and represent the equivalent temperatures at which, under typical indoor conditions, the human body’s thermal balance remains unchanged, assuming the same core and skin temperatures as in the original scenario. The PET index is recommended by the Association of German Engineers (Verein Deutscher Ingenieure – VDI, 2008) as a guideline for climate-sensitive urban and regional planning.

PET index values were calculated for a 35-year-old male weighing 75 kg, assuming a clothing insulation level of 0.50 clo and a fixed metabolic rate of 1.48 met (International Organization for Standardization 2004).

## Results

The results are presented in two sections: (i) the thermal conditions of the entire simulated urban area, and (ii) the influence of courtyard buildings on the thermal conditions of adjacent outdoor spaces and the central courtyard.

### Thermal conditions of the entire simulated urban space

The thermal conditions of the entire simulated urban area were examined using PET maps generated by the post-processing tool Leonardo within ENVI-met, which allowed visualization of the simulation results. This process helped us to identify the differences between central courtyards as a whole and the rest of the urban environment.

**Fig. 3:**
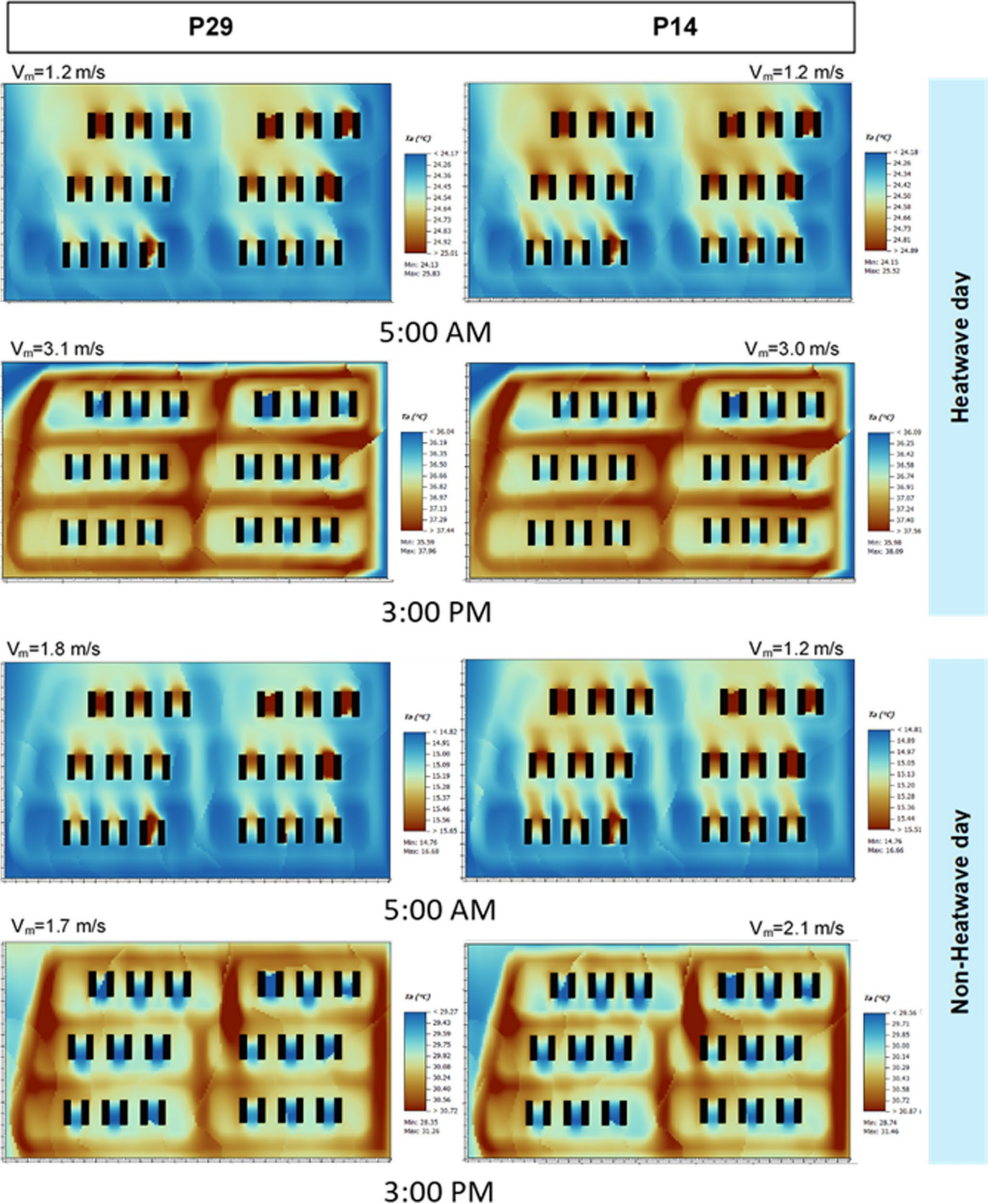
Air temperature in scenarios P29 and P14

Figure 3 shows that, during both heatwave and non-heatwave periods, internal courtyards consistently recorded the lowest daytime air temperatures at 3 pm compared to the rest of the urban area. Under heatwave conditions, temperatures in the courtyards stayed below 36.7 °C, while in the surrounding urban area they ranged from 36.8 °C to 37.9 °C. Additionally, courtyard temperatures varied within a narrow range (36.0–36.7 °C) for both the P29_H and P14_H scenarios, indicating that building height had little impact on daytime thermal conditions during extreme heat. In contrast, non-heatwave periods showed lower overall temperatures in the courtyards at 3 pm, ranging from 28.3 to 29.9 °C in P29_N and 28.7 to 30.2 °C in P14_N. These results suggest that lower building height led to slightly warmer conditions under mild weather.

At night (5 am), the distinction between heatwave and non-heatwave periods was more pronounced. During heatwave events, courtyard temperatures remained elevated, generally between 24 and 26 °C across both building heights, indicating a strong nocturnal heat-retention effect that was largely independent of courtyard geometry. In non-heatwave conditions, early-morning temperatures dropped sharply to approximately 15–16 °C, with minimal variation between the taller and shorter building scenarios. These findings suggest that while building height may slightly influence daytime warming under normal conditions, heatwaves are the dominant factor governing both daytime and nighttime thermal behavior, largely superseding geometric effects.

**Fig. 4:**
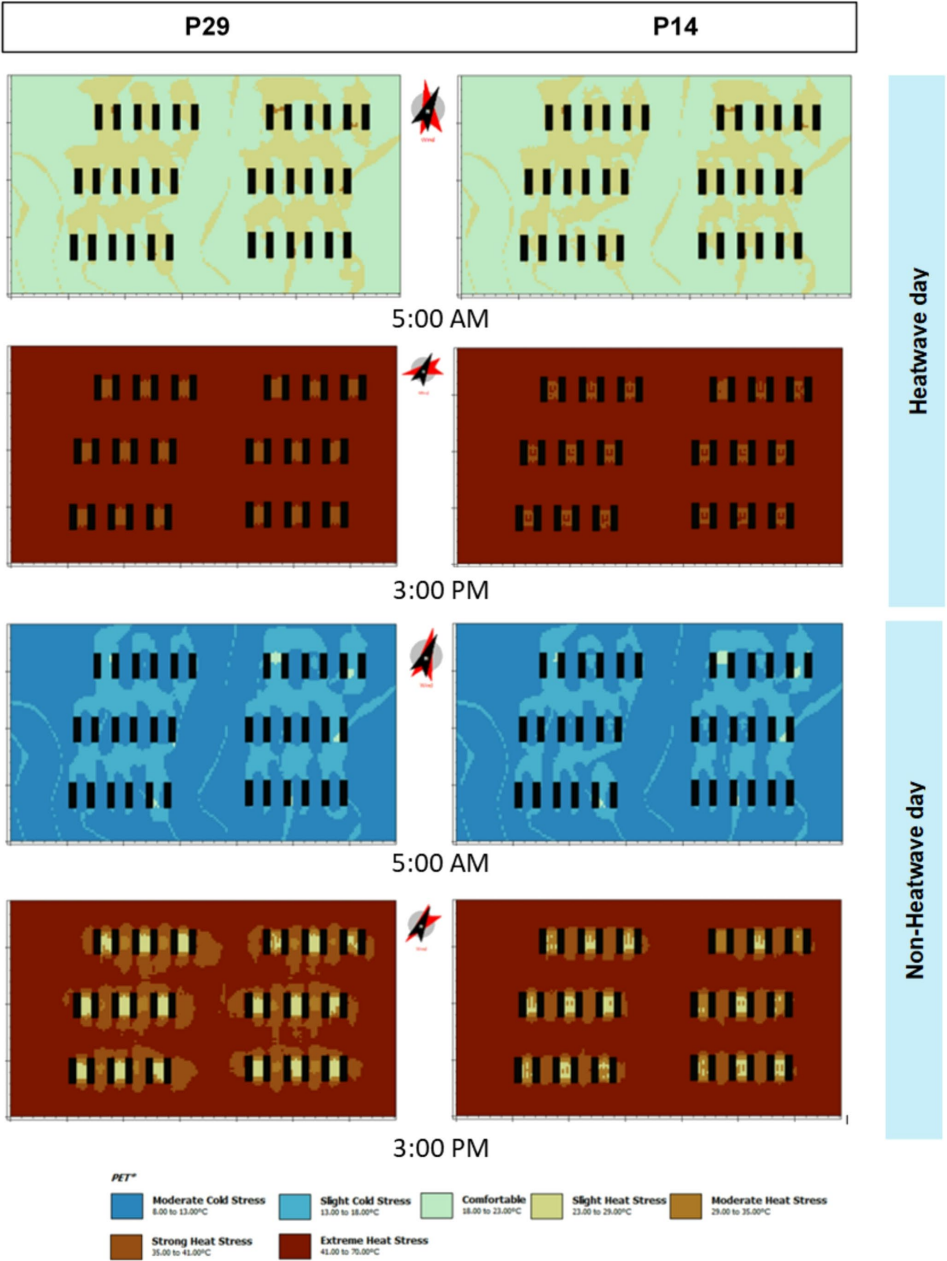
PET in scenarios P29 and P14

In Figs. 3 and 4, the courtyard is depicted by the walls in contact with the ground for a section set at receptor height. The building sections on the north and south sides (as shown in Fig. 2, are not shown at the receptor height of 1.5 m above ground, given the openings, which are equal to the ground-floor height.

Analysis of the PET in the urban environment indicated that, during the heatwave, the entire area experienced high thermal stress. During the day, courtyards experienced lower thermal stress than the surrounding areas, whereas at night their classifications were similar to those of the surrounding areas. Reducing building height from 29 to 14 m decreased nighttime thermal stress but increased daytime stress. On non-heatwave days, courtyards also contributed to reduced daytime thermal stress. At 3 pm, these areas showed mild thermal stress, whereas the rest of the urban area showed strong to extreme thermal stress. As previously observed, reducing building height decreased nighttime thermal stress but increased daytime stress.

Buildings with internal courtyards offer advantages by providing more favorable microclimatic conditions throughout the day than other urban areas. During the heatwave, at 3 pm, patios were the only areas that did not experience extreme thermal stress, indicating their potential as thermal respite for residents. On non-heatwave days, these spaces achieved thermal comfort at noon and only mild stress at 3 pm. In the next section, we will focus on specific points in and around the courtyard (cf. Figure 1 – receptor points in red).

### Influence of courtyard buildings on external thermal conditions

The influence of courtyards on outdoor thermal conditions throughout the day was analyzed by extracting air temperatures at pedestrian level between and within courtyards, as well as the vertical temperature profile along the building height. Additionally, thermal comfort indices, mean radiant temperature, and shading profiles were evaluated.

#### Courtyard shading profile

The shading produced by building courtyards directly influences the microclimate within these spaces. Shading patterns were analyzed using data collected at 8 am, 10 am, 12 pm, and 3 pm (Fig. 3 - **Supplementary materials**).

On September 26, observations indicated that, except for scenario P14 at 12 pm, there was no direct solar radiation at the pedestrian level in the courtyard. Sunlight reached only certain courtyard walls, primarily on the upper floors.

#### Relative comparison between Courtyard and surrounding points in terms of ambient temperature

**Fig. 5:**
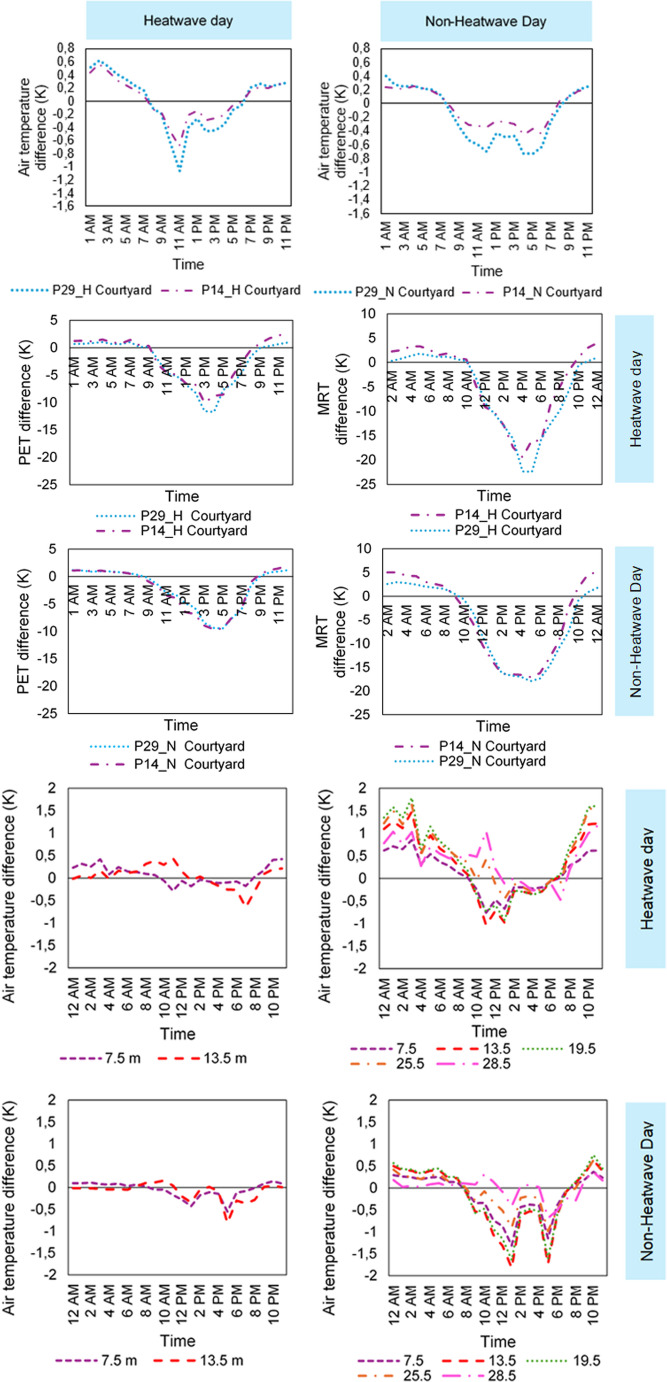
Ensemble with Ta, PET, MRT differences in the courtyards, and vertical relative temperature difference (to 1.5 m – pedestrian height) profile at P14_H (left) and at 29_H (right), on Heatwave and Non-Heatwave days

Figure 5 presents the variations in courtyard air temperature compared to the average temperature in the surroundings of each courtyard typology, using data from both the heatwave day and a non-heatwave day.

During the heatwave, the period of highest solar incidence and peak ambient temperatures corresponded with a noticeable reduction in outdoor air temperature due to courtyard shading. In both scenarios, the most significant reduction occurred at 11 am, reaching − 1.1 K and − 0.7 K, in P29_H and P14_H, respectively. These values indicate that P29_H’s greater verticality enhanced the cooling effect during the day.

At night, courtyard temperatures remained higher than those outside, with both P14_H and P29_H peaking at 2 am and reaching up to 0.6 K in both configurations.

To evaluate heat attenuation during the daytime resulting from shading, as well as radiative cooling at night due to reduced exposure to the sky dome, cumulative negative (daytime cooling) and positive air temperature (Ta) differences (reduced nighttime cooling) were assessed. During the heatwave, the deeper courtyard exhibited a heat attenuation of − 4.1 °C·h, compared to − 2.7 °C·h in P14_H. Heat accumulation in the deeper courtyard was marginally higher than in P14_H, with a difference of − 0.6 °C·h (–4 °C·h versus − 3.4 °C·h). Therefore, daytime cooling is substantially enhanced in P29_H during the heatwave, accompanied by a slight increase in nighttime heat accumulation, compared to P14_H. However, in both cases, heat attenuation comes at the cost of heat accumulating in the courtyards during the night.

On the non-heatwave day, both courtyard configurations exhibited a similar pattern: air temperature decreased during daylight hours and increased during periods with minimal or no solar incidence. Compared with the heatwave scenario, shading dynamics in the courtyards, though not accompanied by extreme temperatures, led to less pronounced reductions in air temperature (Ta) during the midafternoon. The largest decrease was observed at 4 pm in both P29_N and P14_N, with values of − 0.7 K and − 0.5 K, respectively. The greatest increase occurred at 1 am, reaching 0.4 K for P29_N and 0.2 K for P14_N. These findings suggest that increased courtyard verticality contributed to noticeable reductions in daytime temperatures and increases at night. However, the cooling effect was less substantial than that observed during the heatwave.

Under non-heatwave conditions, heat attenuation was more consistent, with higher daytime totals than nighttime heat accumulation and a more pronounced difference between courtyard configurations. Daytime heat attenuation predominated, leading to reduced nighttime heat accumulation and an overall cooling effect in both courtyard types. P29_N exhibited a heat attenuation of − 6.1 °C.h and a heat accumulation of 2.3 °C.h, whereas P14_N showed − 3.5 °C.h and 2.1 °C.h, respectively. Consequently, the deeper courtyard configuration achieved a greater offset of heat relative to heat accumulation during non-heatwave periods.

#### Thermal comfort

The ensemble of graphs in Fig. 5 presents the differences in Physiological Equivalent Temperature (PET) and Mean Radiant Temperature (MRT) inside and outside the courtyards, based on the mean values from the four receptors surrounding the courtyards (refer to Fig. 1 for the receptor scheme). Results are shown for both heatwave and non-heatwave days.

During the heatwave, MRT in the courtyards exhibited greater reductions in the midafternoon, specifically between 4 pm and 5 pm. The largest MRT reductions, which exceeded those observed for air temperature, were recorded on the heatwave day, with decreases of − 22.4 K in P29_H and − 19.4 K in P14_H. In contrast, nighttime increases attributed to stored heat within the courtyards were observed, with maximum increments of 1.8 K in P29_H and 4 K in P14_H. Under heatwave conditions, heat attenuation, measured as MRT offsets, reached − 140 °C·h and − 121 °C·h for P29_H and P14_H, respectively, while nighttime heat accumulation remained relatively low (10.3 °C·h versus 28.9 °C·h). The net effect, calculated as the ratio of heat attenuation to heat accumulation, was 13.6 for P29_H and 4.2 for the P14_H configuration.

Under non-heatwave conditions, temperature reductions in the courtyards were slightly lower than those observed during the heatwave, with values of − 17.9 K in P29_N and − 17 K in P14_N. In contrast, heat attenuation was more pronounced in both configurations, and the effect of courtyard geometry was reduced, resulting in − 145 °C·h and − 140 °C·h in P29_N and P14_N, respectively. As a result, the heat attenuation-to-heat accumulation factor declined to 6.6 and 3.2.

During the heatwave, PET reductions reached − 11.7 K in P29_H and − 10 K in P14_H during the midafternoon. At night, stored heat within the courtyard led to maximum MRT increases of 1.8 K in P29_H and 4 K in P14_H. Patterns of heat stress attenuation and accumulation corresponded to MRT trends, with the deeper courtyard exhibiting greater attenuation than accumulation. Outside the heatwave period, differences between courtyard geometries were less pronounced.

Again, the substantial differences observed in PET values are primarily attributable to the diurnal balance of mean radiant temperature, which is influenced by shading patterns and by the obstruction of the sky dome by courtyard morphology at night.

Figure 4 **(Supplementary materials)** shows that variations in PET stress level classes within the courtyards corresponded with reduced heat stress levels during the heatwave day across the diurnal cycle. A comparative analysis of thermal stress levels between the courtyard receptor and the mean for the receptors outside it indicates that from 1 to 6 pm, both P29_H and P14_H were exposed to strong thermal stress. However, P29_H did not record any hours of extreme thermal stress, whereas P14_H’s courtyard recorded 1 h at this level. Additionally, courtyard areas exhibited a reduction in thermal stress from strong to moderate at 11 am and 12 pm. The impact of heat accumulation is evident in both courtyard geometries, as both recorded fewer hours within the comfort range.

On the day outside the heatwave (Fig. 4- **Supplementary materials)**, moderate and mild cold thermal stress was recorded between the buildings from 1 am to 8 am and at midnight. During the remaining hours, periods of strong, moderate, and mild heat thermal stress were observed. The courtyard areas reduced both cold and heat thermal stress and extended the period within the comfort range by 3 h each. In the P29_N patio, cold thermal stress was reduced from moderate to mild for three hours, and no hours with strong or moderate heat thermal stress were recorded, indicating a reduction in thermal stress for 13 h of the day. In the P14_N patio, the primary difference compared to P29_N was the occurrence of two hours of moderate heat thermal stress. Nevertheless, this courtyard also demonstrated a reduction in thermal stress for 13 h of the day.

PET results suggest that increasing the PET in courtyard areas at night and early morning, when thermal discomfort is caused by cold, can mitigate this issue during those periods. Conversely, in situations where heat is the primary concern, an increase in PET may exacerbate thermal discomfort at night and in the early morning. However, it may provide relief during other times of the day. These findings underscore the potential of central courtyards in buildings to alleviate residents’ thermal stress by offering spaces for social interaction and temporary occupancy.

#### Vertical temperature profile

To analyze the thermal behavior along a vertical profile in both courtyards, temperature measurements were taken at 6 m intervals, starting from pedestrian level (1.5 m, corresponding to the center of gravity of a standing person). Figure 5 presents the temperature variations at different heights within the central area of each courtyard. Temperature differences were calculated relative to the pedestrian level (1.5 m), with negative values indicating a temperature reduction relative to this reference point and vice versa.

Figure 5 shows that, on heatwave day, air temperature in both P14_H and P29_H generally increased with building height at night and when the courtyard internal walls were unshaded. In P14_H at 7.5 m, this increase occurred between 8 pm and 9 am, with the maximum rise of 13.5 m (0.4 K). At this elevation, from approximately 2 pm onwards, temperatures decreased relative to pedestrian level, with the largest negative difference reaching − 0.7 K. Heat stress attenuation and accumulation are more pronounced near ground level, underscoring the courtyard’s moderating effect.

In P29_H, daytime temperature differences relative to the reference pedestrian height generally decrease as building height increases, except within the first 6 m interval. Reduced heat accumulation was observed at 7.5 m, near the ground-floor opening that enhances ventilation, and at 28.5 m, near the building’s top, which also benefits from increased airflow. This produces a nonlinear pattern of daytime heat attenuation with respect to courtyard height. The largest decreases in temperature, reaching − 1 K, and increases, up to 1.8 K, to the reference height were recorded at intermediate heights.

Similar trends are observed on non-heatwave days, although the effects are less pronounced.

The patterns observed during the heatwave suggest that, after periods of solar exposure, the courtyard configuration impeded the dissipation of heat absorbed by the walls, resulting in heat accumulation along the building’s height. The P29 configuration intensified this effect, amplifying both positive and negative thermal gradients at intermediate levels. As previously noted, lower nighttime temperatures on the ground and top floors (28.5 m) can be attributed to increased ventilation at ground level and to higher sky exposure at the uppermost level.

The configuration of the courtyard buildings under analysis likely influenced these results. On the ground floor, the two sides are open, enhancing ventilation and reducing nighttime heat accumulation. In the absence of such openings, ground-floor temperatures may exceed those at higher elevations. Figure 6**(Supplementary materials)** illustrates the ventilation pattern along the height of the courtyard buildings at 2 am, demonstrating a clear difference in wind speed between the ground floor and the upper levels of the building. On the ground floor, average wind speed remains approximately 1.5 m/s, whereas at higher elevations, values approach zero (0 m/s). This pattern persists throughout the heatwave in both P29 and P14. These findings support the interpretation that ground-floor openings enhance local ventilation, thereby reducing heat accumulation in this area.

## Discussion

Thermal conditions in courtyards, encompassing temperature, humidity, wind, and mean radiant temperature, are predominantly influenced by solar radiation, which, in turn, affects thermal sensation (Martinelli and Matzarakis 2017; Rodríguez-Algeciras et al. 2018). The aspect ratio (AR), defined as building height (H) divided by courtyard width (W), is a key parameter. Courtyards with higher AR consistently exhibit a thermal buffering effect. Martinelli and Matzarakis (2017) demonstrated through numerical analysis that a high AR stabilizes courtyard temperatures, with this effect being more pronounced during summer and particularly advantageous in warm climates. Rivera-Gómez et al. (2019), through field monitoring in southern Spain, observed that deep courtyards improve summer microclimatic conditions, with stronger effects at elevated outdoor temperatures.

In this study, the courtyard configuration, characterized by greater proximity to internal walls, influenced air temperature variations. Daytime temperature reductions are attributed to internal shading, while nighttime heat retention is due to limited sky exposure and reduced radiative exchange with the sky dome. A lower sky view factor (SVF) in courtyards decreases daytime solar radiation, resulting in cooler air, but also restricts nighttime long-wave radiation loss and convective heat exchange, leading to elevated nighttime temperatures (Anders et al. 2023; Fan et al. 2024). However, differences in courtyard air temperature compared to adjacent spaces remained minimal during periods of peak heat or radiation, with daytime differences of approximately 1 K during the heatwave.

Callejas and Krüger (2022) demonstrated, through field monitoring of two courtyards with different aspect ratios (ARs) in Cuiabá, Brazil, that both courtyards reduced heat stress during peak daytime hours through their climate-tempering function. The deeper courtyard provided a more consistent cooling effect during the day than the shallower one. Diz-Mellado et al. (2021) monitored two courtyards with distinct ARs in Cordoba, Spain, and found PET attenuation in both, with more pronounced reductions during heatwave peaks. Callejas et al. (2020) observed heat stress attenuation during peak hours in one of the courtyards examined in the present study. The present findings indicate that, with respect to thermal comfort, the reduction in Physiological Equivalent Temperature (PET) within patios during the day and its increase at night can be attributed to the proximity of walls and the courtyard building’s height. This configuration reduces direct solar radiation during the day, resulting in lower temperatures, as shown in Fig. 5. At night, reduced radiative and convective heat losses in the courtyard contribute to heat storage in courtyard surfaces (Anders et al. 2023; Callejas et al. 2020; Fan et al. 2024).

The results concerning the temperature gradient profile underscore the importance of analyzing vertical temperature distributions to understand the internal thermal behavior of courtyards, which directly affects interior building temperatures. M’Saouri El Bat et al. (2023) demonstrated that, in hot climates, square-shaped courtyards tend to increase internal temperatures due to heat accumulation. These findings highlight the need to identify optimal courtyard proportions for different climatic conditions.

Wind patterns also influence the thermal performance of courtyards. Ground-floor openings are essential for enhancing local ventilation. Additionally, the chimney effect can modify stack ventilation, promoting vertical airflow within the courtyard (Chidiadi and Taki 2025), potentially decreasing temperatures with increasing height. However, our results indicated a nonlinear vertical temperature profile, with wind at ground level as a primary factor in reducing local temperatures and heat accumulation on intermediate floors. Future research should evaluate the size and orientation of ground openings relative to the dominant wind direction.

A follow-up study is therefore proposed to build upon this initial investigation into the feasibility of courtyard buildings in social housing projects in Brazil. A parametric study is recommended to examine potential improvements in courtyard thermal performance under the following simulation scenarios:

- Comparisons between a ventilated courtyard and a closed patio;
- Variations in ground-floor opening size and its relation to building height;
- Variations in solar orientation of the courtyard;
- Variations in wind direction and intensity.

The findings of this study may support the inclusion of courtyard buildings among the typologies considered for social housing developments to improve residents’ thermal comfort. From an urban planning perspective, these results highlight the importance of incorporating recommendations on building height and courtyard proportions to maximize this typology’s potential to mitigate thermal stress. Ground-floor openings are also recommended to enhance ventilation and further reduce thermal stress. This approach is particularly relevant for social housing, where climate-sensitive urban design can help reduce heat stress among vulnerable populations. However, further research is required to define the limits of these parameters, given the study’s limitations.

## Limitations

A few limitations should be acknowledged in this study. First, the geometric analysis was limited to two building heights, a single courtyard configuration, a single orientation, and a single ground-floor permeability arrangement. The number of scenarios was further constrained by software limitations, as simulations required substantial computational time. Expanding the analysis to include additional configurations would provide a more comprehensive understanding of the thresholds and contributions of each parameter to thermal stress mitigation. Second, the study focused exclusively on a hot period; future research should examine the performance of courtyard buildings during other seasons, such as winter, and in diverse climatic contexts. Third, the analysis addressed only outdoor thermal conditions. Future studies should also evaluate the impact of courtyard buildings on indoor thermal environments.

Thermal comfort was evaluated using the PET index, based on a standard profile of a 35-year-old male weighing 75 kg, with a clothing insulation level of 0.50 clo and a fixed metabolic rate of 1.48 met (International Organization for Standardization 2004). The specific needs of vulnerable populations, including children, pregnant women, and the elderly residing in social housing projects, were not addressed in detail in this study. However, the combination of social vulnerability and inadequately designed buildings, which typically adhere to standardized housing typologies (Monteiro et al. 2025), further underscores the relevance of this research.

## Conclusions

This study evaluated the thermal performance of high-rise buildings with internal courtyards in social housing developments in São Carlos, Brazil. The analysis included courtyards with ground-floor openings intended to enhance ventilation and provide temporary communal spaces for residents. The findings indicate that these courtyards, by reducing the sky view factor, offered daytime shading and mitigated heat stress. At night, while a slight accumulation of heat was observed, the courtyards reduced thermal stress during periods of cold discomfort. Therefore, buildings with courtyards both attenuated excessive daytime heat and minimized nighttime cold stress.

Across both climatic scenarios, the courtyards maintained more favorable microclimatic conditions than the surrounding environment, providing residents with thermal respite. During heatwaves, all hours of extreme thermal stress were eliminated, with reductions in Physiological Equivalent Temperature (PET) of up to − 11.7 K. In comparison, nighttime temperature increases remained marginal. Analysis of the vertical temperature profile demonstrated that ground-floor openings enhanced ventilation and reduced heat accumulation at that level, whereas intermediate floors experienced greater heat concentration due to limited sky exposure and reduced wind speeds.

A key limitation of this study is its focus on a specific climate and courtyard proportion. Future research should examine a range of climatic contexts, alternative courtyard configurations, and the direct relationship between external thermal performance and the internal comfort of housing units.

In summary, understanding how building height and courtyard morphology influence the urban microclimate is essential for developing climate-resilient housing policies and promoting thermal comfort in socially vulnerable contexts. This issue is particularly significant in countries where large-scale housing initiatives often overlook climate suitability in building design. Additionally, evaluating the impact of urban morphology can inform guidelines for passive building strategies and support the integration of climate-responsive urban morphological indices into master plans and building codes. Such measures can contribute to the development of healthier and more climate-resilient cities.

## Supplementary Information

Below is the link to the electronic supplementary material.

Supplementary Material 1 (DOCX 555 KB)

## Data Availability

The datasets generated and/or analyzed during the current study are not publicly available for undisclosed reasons, but can be made available from the corresponding author upon reasonable request.
