# Supplementary material for: Outdoor thermal comfort in courtyard-shaped housing projects: a simulation study for a tropical region
Source: Int J Biometeorol. 2026 Apr 28;70(5):144. doi: 10.1007/s00484-026-03208-2 (PMC13124780; doi:10.1007/s00484-026-03208-2)
Supplement: Supplementary file 1 — Supplementary Material 1 (DOCX 555 KB) [file 484_2026_3208_MOESM1_ESM.docx]

Supplementary materials

**Figure 1: Climate data for São Carlos – Clima Tool output for 868450_TMYx.2009-2023.**

**Figure 2: Linear regression of simulated and measured temperature.**

**Figure 3: Courtyard shading profile.**


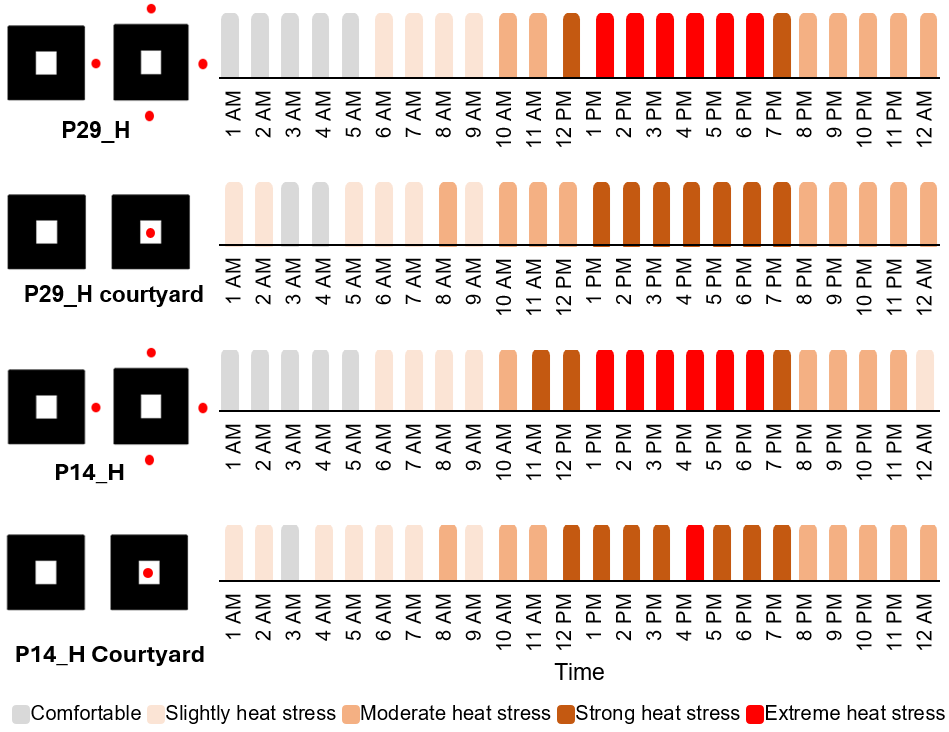


**Figure 4: Heat stress levels inside and outside the courtyards during the heatwave.**

**
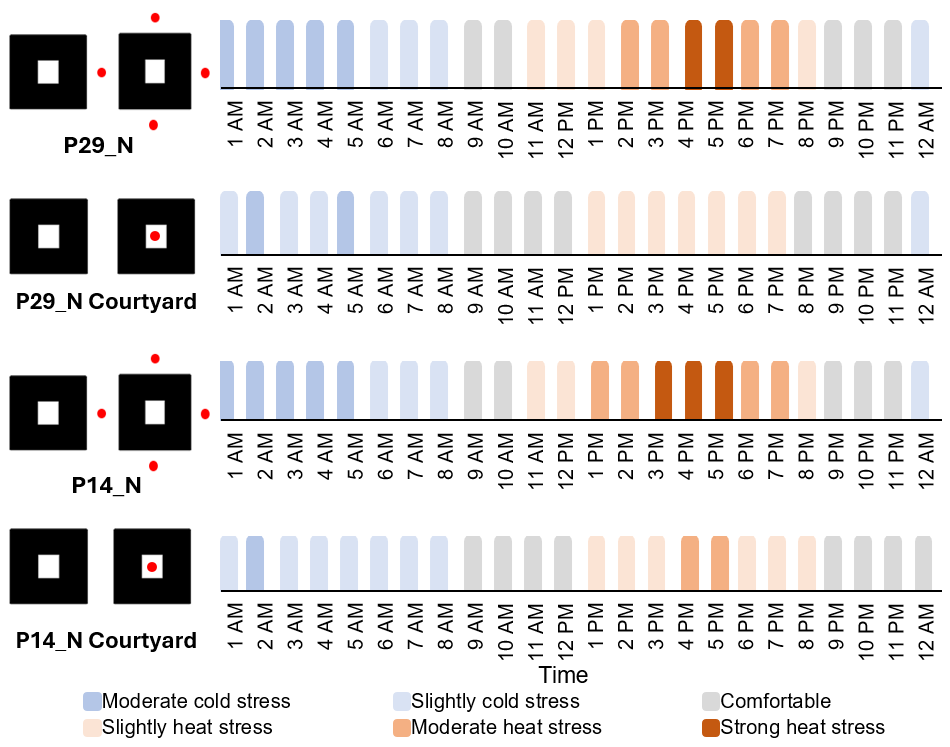
**

**Figure 5: Heat stress levels inside and outside the courtyards outside the heatwave.**

**Figure 6: Vertical profile of air velocity in scenario P29_H, at 2 am.**
